# The Anti-Viral Applications of Marine Resources for COVID-19 Treatment: An Overview

**DOI:** 10.3390/md19080409

**Published:** 2021-07-23

**Authors:** Sarah Geahchan, Hermann Ehrlich, M. Azizur Rahman

**Affiliations:** 1 Centre for Climate Change Research, Toronto, ON M4P 1J4, Canada; sarah.geahchan@mail.utoronto.ca (S.G.); Hermann.Ehrlich@esm.tu-freiberg.de (H.E.); 2 Department of Pharmacology and Toxicology, University of Toronto, Toronto, ON M5S 2E8, Canada; 3 A.R. Environmental Solutions, University of Toronto, ICUBE-UTM, Mississauga, ON L5L 1C6, Canada; 4 Institute of Electronic and Sensor Materials, TU Bergakademie Freiberg, 09599 Freiberg, Germany; 5 Center for Advanced Technology, Adam Mickiewicz University, 61614 Poznan, Poland

**Keywords:** Sars-Cov-2, COVID-19, marine protein, antiviral, bromotyrosines, marine sponge, marine algae, phycocyanobilins, sulfated polysaccharides

## Abstract

The ongoing pandemic has led to an urgent need for novel drug discovery and potential therapeutics for Sars-CoV-2 infected patients. Although Remdesivir and the anti-inflammatory agent dexamethasone are currently on the market for treatment, Remdesivir lacks full efficacy and thus, more drugs are needed. This review was conducted through literature search of PubMed, MDPI, Google Scholar and Scopus. Upon review of existing literature, it is evident that marine organisms harbor numerous active metabolites with anti-viral properties that serve as potential leads for COVID-19 therapy. Inorganic polyphosphates (polyP) naturally found in marine bacteria and sponges have been shown to prevent viral entry, induce the innate immune response, and downregulate human ACE-2. Furthermore, several marine metabolites isolated from diverse sponges and algae have been shown to inhibit main protease (M^pro^), a crucial protein required for the viral life cycle. Sulfated polysaccharides have also been shown to have potent anti-viral effects due to their anionic properties and high molecular weight. Likewise, select marine sponges produce bromotyrosines which have been shown to prevent viral entry, replication and protein synthesis. The numerous compounds isolated from marine resources demonstrate significant potential against COVID-19. The present review for the first time highlights marine bioactive compounds, their sources, and their anti-viral mechanisms of action, with a focus on potential COVID-19 treatment.

## 1. Introduction

**Figure 1:**
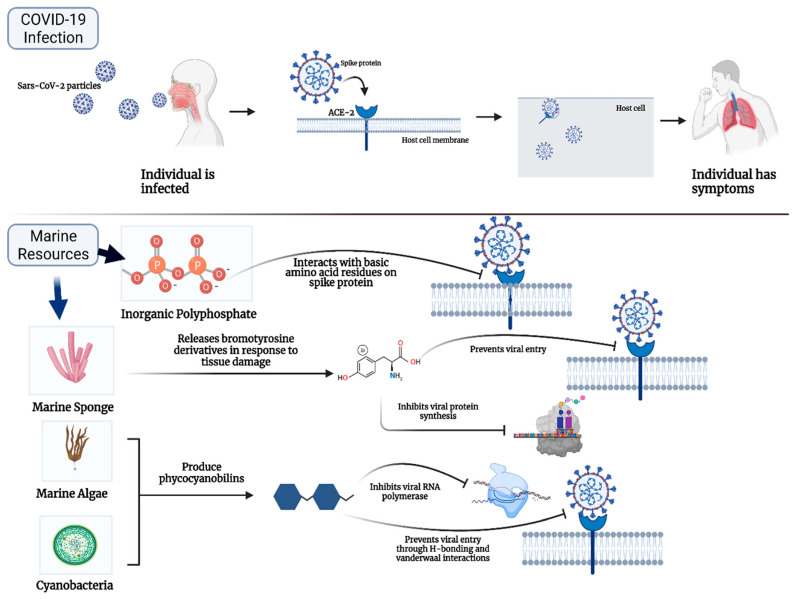
Overview of Sars-CoV-2 infection and various marine compounds that have anti-viral properties. Top half depicts the process of Sars-CoV-2 Infection. Viral particles enter the individual’s airways, where the spike proteins bind to human ACE-2 receptors on the surface of our cells. In this way, the viral particles enter the host cell causing an inflammatory response and manifesting symptoms. Bottom half depicts how several marine resources have anti-viral properties serving as promising therapeutic resources. [Created with BioRender.com accessed on 21 June 2021].

COVID-19 is an infectious respiratory disease caused by the newly identified strain of coronavirus, Sars-CoV-2 [1,2,3,4]. This single stranded RNA virus can infect the respiratory tract by binding to ACE-2 protein receptors on the surface of host cells [1,2,4] (Figure 1). The viral particles have spike proteins on their surface which contain a receptor binding domain (RBD) that is recognized by the human ACE-2 receptor [2]. This unique RBD specifically binds to a lysine residue on the ACE-2 receptor, making the RBD a promising pharmacological target [2,4]. By infecting the airways and lungs, the viral particles initiate an inflammatory response in the body, damaging the host tissue [3,4]. This can lead to end stage respiratory disease, systemic involvement, and eventual death. Although the utility of COVID-19 vaccines has been effective in preventing infection, control cannot depend on vaccines, rather treatments are needed as well [5]. 

Currently, standard therapy includes Remdesivir in combination with the anti-inflammatory agents’ dexamethasone or baricitinib [6,7,8,9,10]. Remdesivir is an adenosine analog prodrug that is able to inhibit viral RNA dependent RNA polymerases [8,10,11,12]. However, the literature provides contradicting evidence on the efficacy of Remdesivir. For example, a randomized, double- blind clinical trial demonstrated that patients treated with Remdesivir over a 10-day period recovered significantly faster than placebo (11 days vs. 15 days) [11]. Similarly, Spinner et al. performed an open-label randomized clinical trial and found that when administered a 5-day course of Remdesivir, patients suffering from moderate COVID-19 had significantly better clinical status after 11 days in comparison to placebo (*p* = 0.02) [12]. Contrarily, patients who were administered a 10-day course of Remdesivir displayed no statistically significant difference on day 11 compared to placebo (*p* = 0.18). Furthermore, Goldman et al. demonstrated that there was no significant difference in a 5-day vs. 10-day course of Remdesivir in patients with severe COVID-19. Using an ordinal scale to assess clinical improvements, 64% of patients in the 5-day group and 54% in the 10-day group improved by 2 points [8]. However, this study was limited in that it did not have a placebo control to assess the magnitude of benefit. Likewise, Pan et al. showed that Remdesivir fails to improve mortality outcomes. Death occurred in 10.95% of patients receiving Remdesivir and 11.19% of patients receiving its control (*p* = 0.50) showing that Remdesivir is not an efficacious drug [10]. Furthermore, several studies reported adverse effects of Remdesivir including nausea, worsening respiratory failure, constipation, hypokalemia and headaches [8,11,12]. Thus, these several contradicting results along with the adverse effects demonstrate that there is an urgent need for the development of novel drugs for efficacious COVID-19 treatment.

Favorably, marine resources harbor many valuable micro and macro-organisms that produce compounds with pharmacological potential [13,14,15]. Marine resources are advantageous in that they are environmentally friendly, have minimal toxins and are metabolically compatible [16]. Being rich and diverse, marine resources have anti-bacterial, anti-cancerous, anti-inflammatory, and anti-viral properties that make them critical sources of pharmacological targets [13,14,15,16,17]. The anti-viral aptitude of marine organisms makes them promising for therapeutic use in treatment of COVID-19. Numerous marine resources have shown to be effective in treating other RNA viruses, such as Human Immunodeficiency virus (HIV) and Influenza virus. For example, the marine algae, *Padina tetrastromatica* has shown to have immune stimulatory, antioxidant and anti-HIV properties [17]. Similarly, portimine, a molecule from the dinoflagellate *Vulcanodinium rugosum*, has shown to have significant anti-HIV effects by directly inhibiting the reverse transcriptase enzyme [18]. Furthermore, rhamnan sulfates derived from marine Green Algae *Monostroma nitidum* have been shown to inhibit the replication of influenza virus [19]. HIV and influenza are single stranded RNA viruses, like COVID-19, thus marine organisms are promising for treatment against Sars-CoV-2.

In fact, numerous natural compounds from marine resources are currently being investigated for potential anti-viral effects against COVID-19. Specifically, natural inorganic polyphosphate (polyP) from marine bacteria and sponges has been shown to have protective effects against COVID-19 [20,21,22,23,24,25]. Several studies have demonstrated its ability to bind the spike protein on the viral particles and inhibit interaction with ACE-2 (Figure 1), as well as induce the degradation of ACE-2 on host cells. In addition, polyP has been shown to have synergistic anti-viral effects in combination with the anti-inflammatory agent dexamethasone or the anti-oxidant compound quercetin. Furthermore, many studies have shown that several marine metabolites isolated from scleractinia related organisms, sponges and algae can interact with the main protease of Sars-CoV-2, M^pro^ [13,14,15,26,27,28]. M^pro^ is a crucial protein enzyme of the virus and has a critical role in mediating the replication and transcription of the viral particles, making it a potential drug target against the virus [27,28,29]. As depicted by Figure 1, several marine metabolites such as phycocyanbillins were found to bind to RNA dependent RNA polymerase (RdRp) with equivalent or higher potency than Remdesivir, making them advantageous over standard therapy [27,28,29,30,31,32,33,34,35]. Notably, marine organisms are not limited in the production of only one compound. For example, in addition to harboring polyP and other metabolites, marine sponges produce the valuable compound, bromotyrosine, in response to tissue damage from the environment (Figure 1). These bromotyrosines have shown to have significant anti-HIV, anti-cancerous and anti-bacterial affects and are currently being studied for potential COVID-19 treatment [36,37,38,39,40,41,42]. Thus, it is evident that marine resources harbor an enormous pool of compounds that are favorable for further development to potentially treat Sars-Cov-2 infected patients. This review provides a summary and overview of the various marine resources and their promising potential for Sars-CoV-2 treatment.

## 2. Marine Natural Polymer: Inorganic Polyphosphate for COVID-19 Treatment

Inorganic polyphosphate (polyP) is a compound ubiquitously expressed in every cell, including marine organisms, like the cyanobacterium synechcoccus [20,21,22,23,24]. PolyP is found abundantly in marine bacteria, sponges as well as human blood platelets [20,21,22,23,24,25]. PolyP, which is released from platelets, interacts with the protease coagulation factor VII and plays an important role in the mediation of blood clots [20,21,22,23,24,25]. It has been shown that COVID-19 patients have deficient platelet counts and as a result, have reduced polyP in addition to chemical immune mediators such as cytokines and chemokines [20,21]. Since polyP is abundantly present in platelets, a reduction or deficiency in platelets causes significant reduction in polyP, which can lead to problems with coagulation in Sars-CoV-2 patients [20,21,22]. Asymptomatic Sars-CoV-2 patients do not have a severe platelet deficiency and thus, it has been proposed that polyP serves a protective role in these patients. PolyP has been shown to bind the RBD of the spike protein on Sars-Cov-2 particles through its basic residues and prevent the binding of the spike protein to host ACE-2 receptors [20,21,22,23,24] (Refer to Table 1). 

In particular, one study synthesized a model depicting the proposed mechanism by which polyP interacts with the spike protein [24]. Approximately 15 phosphate units of polyP are thought to interact with the basic residues, Arg, Lys and His on the spike protein [20,24]. Furthermore, the study found that the soluble polyP significantly inhibited the interaction of the spike protein and Ace-2 at concentrations ranging from 1 μg/mL to 100 μg/mL [24]. This inhibition was found to be 70% effective, suggesting that polyP has protective anti-viral effects [24]. Notably, during 24 h of incubation, polyP ranging up to 100 μg/mL had no toxic effect on cells.

**Figure 2:**
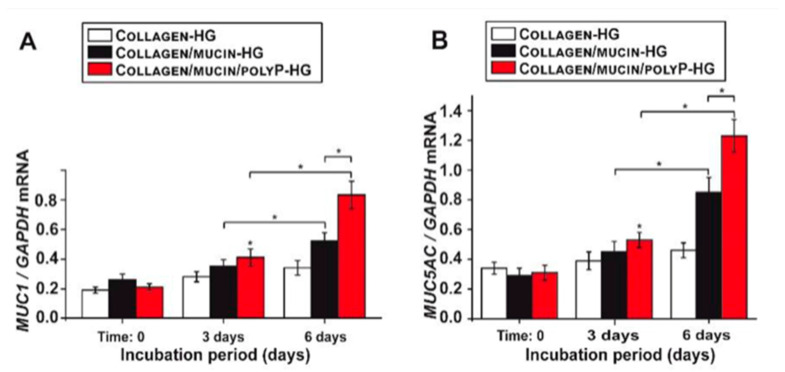
Expression of MUC1 and MUC5AC genes in A549 cells over 6 days. (**A**) Relative expression of MUC1 incubated for 3 and 6 days. (**B**) Relative expression of MUC5AC incubated for 3 and 6 days [21].

Further, polyP is typically hydrolyzed by alkaline phosphatase (ALP) which releases free energy and results in the formation of ADP which becomes phosphorylated to form ATP [21,22]. It has been found that polyP is able to stimulate the innate antiviral immune response by inducing mucin gene expression through the increased generation of ATP [21,22]. As seen by Figure 2, compared to the controls (black and white), when polyP was added, mRNA levels of MUC1, a main mucin type, significantly increased over 6 days compared to controls [21]. These findings suggest that in addition to preventing Sars-Cov-2 attachment to host cells, polyP boosts the innate immune system and mucosal defense against the virus. This is significant as one’s status of innate immunity drastically contributes to the manifestation of COVID-19 in patients.

**Figure 3:**
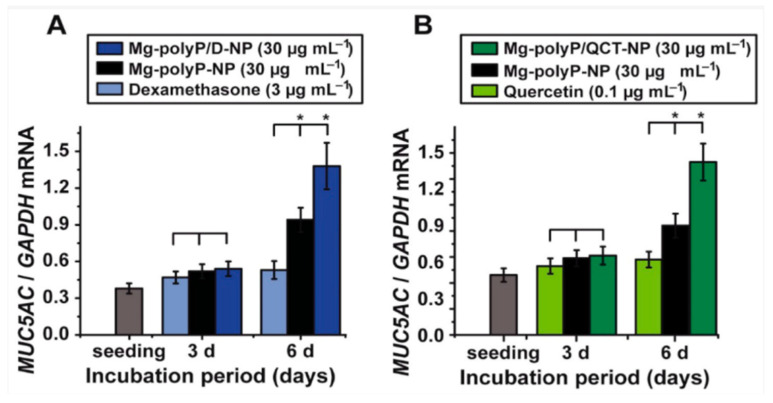
Expression of MUC5AC genes in A549 cells. (**A**) Cells were exposed to 3 μg mL^−1^ of dexamethasone, 30 μg mL^−1^ of polyP and dexamethasone (“Mg-polyP/D-NP”) or 30 μg mL^−1^ of polyP (“Mg-polyP-NP”). (**B**) Cells were exposed to 0.1 μg mL^−1^ of quercetin, 30 μg mL^−1^ of polyP and quercetin (“Mg-polyP/QCT-NP”) or 30 μg mL^−1^ of polyP (“Mg-polyP-NP”) [22].

Similarly, another study utilized nanoparticles of polyP in addition to dexamethasone or the metabolite quercetin on A459 epithelial cells [22]. The study found that polyP increased the expression of a major mucus glycoprotein MUC5AC [22]. As seen in Figure 3, polyP combined with 4.5 μM dexamethasone (anti-inflammatory) or 0.08 μg quercetin (antioxidant) significantly increased the expression of MUC5AC than either drug alone. Dexamethasone causes some toxic effects (induction of apoptosis) at concentrations greater than 100 mM, thus the 4.5 μM used in the study did not affect cell viability. Similarly, quercetin is cytotoxic at concentrations greater than 0.3 μg/mL, thus the 0.08 μg used in the study did not impact cell viability [22]. These synergistic effects demonstrate the potential of combinatory therapy involving both marine resources and non-marine drugs. MUC5AC and MUC1 genes encode for proteins that play an important role in the mucosal barrier and are shown to be elevated in healthy individuals in comparison to Sars-Cov-2 patients [22]. This is significant as the mucosal barrier is a critical part of defense against Sars-Cov-2 and other pathogens, as it clears the viral particles and dictates the accessibility of the pathogens to the host epithelial cells.

In line with these studies, Ferrucci et al. demonstrated that polyP120 binds to the ACE2 receptors and downregulates it by inducing its degradation [25]. As seen by Ferrucci et al., immunoblotting demonstrated a dose-dependent decrease in ACE-2 expression as the concentration of polyP120 increased over 24 h [25]. This is promising as the mode of Sars-Cov-2 entry is through the ACE2 receptor on host cells. Furthermore, the study demonstrated that polyP120 impairs the synthesis of viral proteins required for the replication of Sars-Cov-2 by impairing viral transcription and replication [25]. The study also found that the inorganic polyphosphate inhibits the Nf-kB pathway and thus, reduces the cytokine storm typically associated with COVID-19 infection [25]. This is valuable as systemic infection and significant inflammatory immune response can be detrimental.

## 3. Promising Compounds from Marine Algae, Bacteria, Sponges, and Fish for COVID-19 Treatment

**Figure 4:**
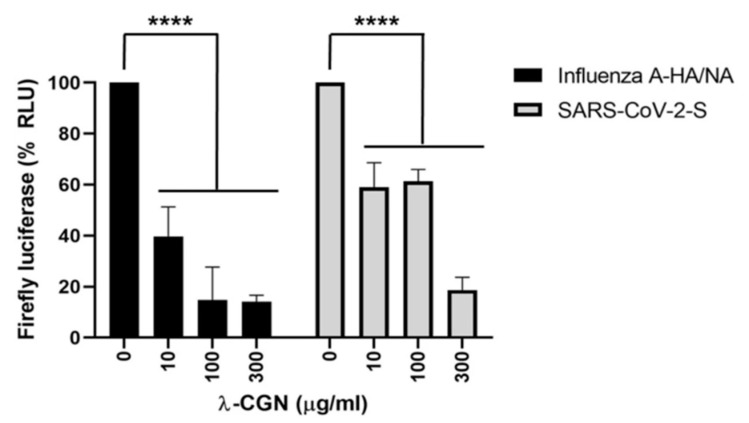
Inhibition of Influenza A and Sars-CoV-2 by lambda-carrageenan expressed as firefly luciferase. Influenza A viral proteins (black bars) over increasing concentrations of lambda-carrageenan. Sars-Cov-2 spike proteins (grey bars) over increasing concentration of lambda-carrageenan [43].

Marine algae are known to be a source of numerous bioactive substances such as vitamin E, B12, phycocyanin, lutein and polysaccharides [48,49,50]. Specifically, lambda-carrageenan is a polysaccharide purified from marine red algae (Refer to Table 1) and has anti-viral, anti-bacterial, anti-cancerous and anti-coagulant functions [48,49,50]. It has been shown to effectively inhibit both influenza virus and Sars-Cov-2 [43]. A study done by Jang et al. demonstrated that the marine polysaccharide was able to reduce the expression of viral proteins and suppress viral replication dose-dependently [43]. Depicted in Figure 4, as the dose of lambda-carrageenan increased from 0 to 300 μg/mL, the presence of spike viral proteins on Sars-CoV-2 and influenza A viral proteins significantly decreased [29]. Inhibition of Influenza virus and Sars-Cov-2 depicted EC50 values 0.3–1.4 μg/mL and 0.9 ± 1.1 μg/mL, respectively. Favorably, no host cell toxicity was observed at concentrations up to 300 μg/mL [29]. The study also found that mice challenged with Sars-Cov-2 virus and later given Lambda-carrageenan had a 60% survival rate, as the polysaccharide inhibited viral entry and viral replication [43]. These findings reveal the anti-viral properties of lambda-carrageenan and make it a promising marine resource for COVID-19 treatment. 

Although these findings are promising, it is important to mention potential adverse effects of lambda-carrageenan. Previous studies have reported the oligosaccharides derived from the carrageenan family (kappa- and lambda-carrageenan) can impair blood vessel development by inhibiting the growth of new blood vessels [48,51]. It was also found that at 200 μg/mL they could inhibit migration, proliferation as well as tube formation of human umbilical vein endothelial cells [48,51]. These results demonstrate potential toxic effects to humans; however, more in vitro and in vivo toxicology studies are needed. It is important these studies be taken into consideration for further development of lambda-carrageenan against Sars-Cov-2.

The pharmacological potential of sea organisms is further prevalent in scleractinia associated organisms such as bacteria and fungi [13,14,15,26,27,28,29]. These organisms are known to produce a variety of metabolites making them implicated in inflammation and viral infection [13,14,15,26,27,28,29]. In a study done by Zahran et al., scleractinia related metabolites were analyzed, and molecular docking was performed to determine potential Sars-CoV-2 anti-viral effects [29]. It was found that two specific microbial metabolites (Terphenyllin and Tirandamycin A) form hydrogen bonds and dock with high affinity to the main protease (M^pro^) [29]. These marine metabolites are thought to be promising leads for inhibition of the main protease, which plays an important role in the life cycle of the virus. Similarly, Gentile et al. identified seventeen potential M^pro^ inhibitors from the class phlorotannins isolated from *Sargassum spinuligerum* brown algea [30]. Docking energies ranged from −14.6 to −10.7 kcal/mol and the compounds interacted with M^pro^ through extensive hydrogen bonding as well as hydrophobic interactions. In addition, the Sars-Cov-2 RNA polymerase along with nsp7/8 are required for the RNA replication and viral protein synthesis [30]. Remdesivir is a known inhibitor of the RNA dependent-RNA polymerase, and three marine Scleractinia metabolites were found to bind the polymerase in the same position as Remdesivir [6,7,8,30]. This finding suggests that these marine metabolites are promising leads for the inhibition of viral replication and thus, treatment of COVID-19.

**Figure 5:**
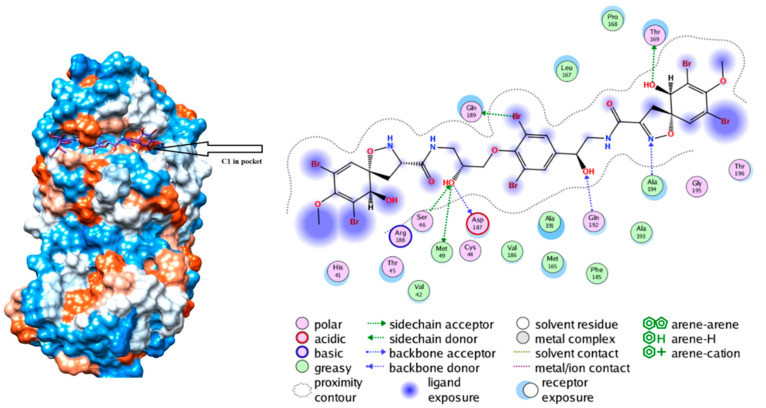
Depicts the interaction of C1 compound with Sars-Cov-2 M^pro^ after molecular dynamic stimulation. C1 hydrogen bonds with Ser46, Met49, Asp186, Gln192, Ala194, Thr169 and Gln189. Reproduced with permission from Khan, M.T. et al., Marine natural com-pounds as potents inhibitors against the main protease of SARS-CoV-2-a molecular dynamic study; published by Taylor & Francis, 2020, [31].

Moreover, a study done by Khan et al. performed molecular docking analysis on M^pro^ and found that several marine compounds demonstrated promising binding interactions [31]. Five marine compounds, isolated from sea sponges of family Aplysinidae and *Petrosia stronglyophora* sp. and the soft coral *Pterogorgia citrina* were found to interact with M^pro^ through hydrogen and hydrophobic interactions [31]. Analysis of ADME properties depicted them to have potential Sars-Cov-2 therapeutic application [31]. One marine compound (C1, from the family Aplysinidae) was found to fit the M^pro^ pocket the best, having affinity for all regions of M^pro^ with much higher hydrogen and hydrophobic interactions [31]. As seen in Figure 5, C1 residues Ser46, Met49, Asp186, Gln192, Ala194, Thr169 as well as Gln189 interacted with M^pro^ through H-bonding [31]. This finding gives insight into the spatial location that the compounds have in the binding pocket where there are also hydrophobic and electrostatic interactions present. 

Additionally, phycocyanobilins (PCBs) are pigment compounds present in some types of cyanobacteria as well as the algae rhodophytes [32,33,44]. They have been shown to have antioxidant as well as anti-viral properties making them promising leads for COVID-19 therapy. One study done by Pendyala and Patras demonstrated that the marine PCB among others, are potent inhibitors of M^pro^ and RNA dependant RNA polymerase (RdRp) of Sars-Cov-2 [32]. Through in-silico screening, it was depicted that the PCBs had a higher binding affinity to RdRp than the current drug Remdesivir which signifies the potential these compounds have for anti-Sars-Cov-2 effects [32]. Similarly, Petit et al. performed an in-silico study and found that PCB among other phycobilin compounds expressed by *Arthrospira* had promising anti-viral properties against Sars-Cov-2 [33]. The study found that PCB interacted with the RBD of the virus’ spike protein through Vander Waal interactions as well as hydrogen bonding. PCB was found to have a competitive binding energy of −7.2 kcal/mol suggesting it to be a potential anti-viral compound [33]. Promisingly, the study reported phycobilin compounds from *Arthrospira* to have minimal to no cytotoxicity to cells and was shown to be effective at low doses (1–10 μg/mL). PCBs were reported to have low mutagenicity, carcinogenicity and reprotoxicity [33]. These findings demonstrate that PCBs have substantial anti-viral effects and may serve as promising agents against Sars-Cov-2.

The resources marine organisms provide are never ending. Cyanobacteria harbor numerous metabolites such as sulfated polysaccharides which are known to have anti-viral properties [34]. Sulfated polysaccharides have anti-viral activity against HSV, hepatitis B virus as well as retroviruses [34,35,45]. They have been shown to play an important role in shielding against the virus due to their anionic features as well as molecular weight which together are able to have anti-viral effects [52]. Due to these anti-viral abilities, it is proposed that the polysaccharides harbor large potential against Sars-Cov-2 [34,35,45,46]. In fact, a study done by Kwon et al. demonstrated that a specific type of sulfated polysaccharide, Fucoidan, from *Saccharina japonica*, had anti-viral activity against Sars-Cov-2 [35]. The study showed that the marine compound was more potent than Remdesivir, suggesting that it is a promising therapeutic agent against COVID-19 [35]. Similarly, a study done by Song et al. demonstrated that fucoidan from brown algae, cucumber sulfated polysaccharide as well as carrageenan from red algae all displayed anti-viral properties at concentrations ranging from 3.9 to 500 μg/mL [45]. It was found that the cucumber sulfated polysaccharide had the strongest inhibitory effects due to its ability to bind the spike protein and inhibit viral entry into cells [45]. Favorably, at concentrations up to 500 μg/mL, no cytotoxicity was observed as depicted by no significant changes in cell viability [45]. These findings depict the potential that sulfated polysaccharides have for effective treatment of Sars-Cov-2.

As recently proposed by Nguyen and colleagues, “marine sponges have the capacity to filter large volumes of ‘virus-laden’ seawater through their bodies and host dense communities of microbial symbionts, which are likely accessible to viral infection” [53]. These organisms with typical filter-feeding lifestyles are constantly in contact with the surrounding environment where one milliliter of water can contain up to 10 million viruses that move through their body per day [53]. Marine *Halichondria panicea* demosponge has been shown to rapidly filter out viruses at high rates (176 mL h^−1^ g tissue dry wt^−1^) [54]. Thus, it is not surprising that investigations on viral ecogenomics across the four sponge classes is in trend [55]. Despite predation [56], sponges develop highly specialized chemical defense mechanisms based on the biosynthesis of effective antiviral biomacromolecules [57,58]. Such antiviral substances of poriferan origin such as nucleoside Ara-A (vidarabine) isolated from sponge *Tethya crypta* [59], or spongouridine and spongothymidine [60], have been recognized recently. Sponge extracts which have been shown to be active against human adenovirus (HAdV) have also been reported [61]. After 2019, special attention has been payed to poriferan inhibitors of SARS-CoV-2 key target proteins (i.e., Ilimaquinone) [36], or Remdesivir [37].

**Figure 6:**
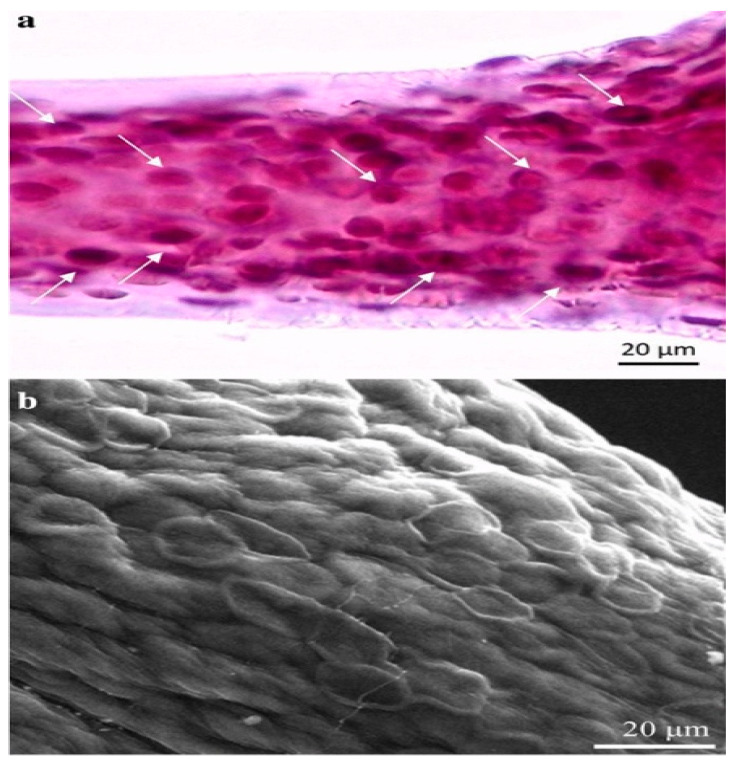
Image depicts the specialized spherulocyte cells in the chitinous skeletal fibers of verongiid sponges. (**a**) Light microscopy image showing the distribution of these specialized spherulocyte cells (arrows). (**b**) Scanning lectron microscopy represents the morphology of these cells. Reproduced with permission from Muzychka, L. et al., Marine biomimetics: Bromotyrosines loaded chitinous skeleton as source of antibacterial agents; published by Springer Nature, 2021, [47].

Marine demosponges, which belong to the Verongiida order, are recognized producers of bromotyrosines [38,39,40]. They possess anti-viral, anti-bacterial and anti-parasitic properties [40,41,42,46]. These brominated compounds in the aplysinidae family of verongiids are produced within specialized cells known as spherulocytes (Figure 6) [47]. These cells are sensitive to changes in the environment and can release bromotyrosine in response to environmental stimulus [47].

Recently, Muzychka and co-workers isolated the bromotyrosine derivative 3,5-dibromoquinolacetic acid using a novel biomimetic water-based method and found it to have anti-microbial properties against selected clinical stains of *Staphylococcus aureus*, *Enterococcus faecalis* and *Propionibacterium acnes* [47]. The study also showed that Aeroplysinin-1 and 2 also had anti-microbial properties [47]. Similarly, another study was able to isolate the bromotyrosine Aeroplysinin-1 and found that it was able to decrease the viability of neuroblastoma cell lines and inhibit the growth of drug resistant bacteria [41]. Similarly, bromotyrosines have been shown to have potential anti-tumor and anti-metastatic affects displaying cytotoxic properties [40]. Dreschel et al. investigated the potential cytotoxicity and therapeutic window of two bromotyrosine derivatives, Aerothionin and Homoaerothinin [40]. The study found that after treating mouse endothelial cells with 25 to 50 μM Aerothionin or Homoaerothinin, cell viability was significantly reduced. However, the bromotyrosine derivatives had efficacious anti-cancerous effects at 10 μM of Aerothionin or Homoaerothinin, with minimal cytotoxic effects [40]. 

Moreover, bromotyrosines have also been found to inhibit HIV-1 infection through inhibiting protein synthesis, replication and proliferation of the virus as well as preventing its entry into host cells (refer to Table 1) [42]. This is promising as bromotyrosines may be useful for treating COVID-19. In a recent press release, Dr. Ehrlich emphasized that his research group was able isolate reasonable amounts of bromotyrosines which can be used to further investigate their potential against the Sars-Cov-2 virus [46]. Thus, given bromotyrosines anti-viral and anti-pathogenic effects, future clinical trials investigating it further are valuable for potential COVID-19 treatment. 

## 4. Promising Advantages and Limitations of Marine Resources

The resources marine organisms harbor are limitless and consistently prove efficacious at combatting viruses, bacteria, cancers and other pathogens. Their unique chemical structures and diversity introduce novel mechanisms of action, making them especially valuable against drug-resistant pathogens. Some marine compounds that do share similar mechanisms of action with other known approved drugs have shown to be more potent. As discussed above, PCBs and sulfated polysaccharides have shown to bind and inhibit RdRp with higher affinity than current standard therapy Remdesivir [27,30]. Furthermore, each marine compound serves not one single role, rather multiple roles proving valuable for different applications. For example, the PCBs are not only able to inhibit RdRp, but also interact with the RBD of the viral spike proteins, making them even more advantageous over Remdesivir [33]. In addition, majority of the compounds such as sulfated polysaccharides do not only possess anti-viral properties, but also anti-coagulant, anti-inflammatory, anti-oxidant and anti-bacterial [62]. These multi-faceted properties of marine compounds make them very efficacious agents against Sars-Cov-2. This is advantageous over synthetic compounds which typically possess one single valuable property and are often administered in combinatory therapies, which increases the possibility of drug-drug interactions. In addition, due to the abundance and diversity of marine resources, they are highly cost-effective. This makes them valuable, as the current standard treatment Remdesivir costs approximately $2600 for a 5-day course treatment [63]. Furthermore, at effective concentrations of polyP (<100 μg/mL), lambda carrageenan (<300 μg/mL), PCBs (<10 μg/mL), sulfated polysaccharides (<500 μg/mL) and bromotyrosines (10 μM), no toxic effects on cells were observed [21,33,40,43,45].

However, the process of marine drug development is faced with many challenges. Firstly, although the sea harbors countless organisms, accessibility to majority of these resources are limited [64]. Although plentiful compounds are accessible close to shore, there remain other regions of the ocean that likely possess unknown organisms and thus, new therapies [64]. Furthermore, to continue the development of promising compounds through pre-clinical and clinical trials, there must be a continuous supply of the compounds. This presents a challenge as large-scale production may harm the marine ecosystem [64,65]. Fortunately, rapid technological advancements in synthetic chemistry and biotechnology provide a potential solution to this problem [64]. In addition, many potential anti-viral metabolites have only been tested in vitro or visualized through molecular docking assays. More in vivo studies are needed to further investigate potential adverse effects and drug delivery requirements. Despite the challenges faced, it is clear that marine organisms serve as a promising avenue for future pharmacological intervention.

## 5. Conclusions

The present review highlights the current research on marine resources and their anti-viral bioactive metabolites. Marine organisms and the compounds they synthesize are profoundly valuable for COVID-19 treatment. PolyP has shown to effectively inhibit the RBD of the spike protein, and as a result, inhibit its ability to bind ACE-2 on host cells. This has substantial potential in preventing infection in Sars-CoV-2 patients. The compound is further promising in that it can stimulate the immune response and thus have an immune-protective function in patients. In addition to polyP, various other compounds such as PCBs, sulfated polysaccharides and bromotyrosines have also been shown to have anti-viral effects, which make them promising agents for further development into COVID-19 therapeutics. Overall, the marine waters are full of micro and macro-organisms that harbor extensive amounts of metabolites, most of which have not yet been discovered. Thus, investigating and discovering novel resources that come from the sea bring promising potential therapeutics for treating patients with COVID-19.

## Figures and Tables

**Table 1:** Summary of marine compounds for potential Sars-Cov-2 treatment.

Marine Compound	Source	Mechanism of Action
Inorganic polyphosphate (polyP) [21,22,24,25]	Marine sponges, bacteria (ex. Cyanobacterium synepchcoccus)	- Binds RBD of spike protein and prevents binding to ACE-2 - Stimulates innate immune system through upregulation of mucosal proteins - Synergistic effects with 4.5 μM dexamethasone or 0.08 g quercetin - PolyP120 downregulates ACE-2 by inducing its degradation - PolyP120 inhibits Nf-kB pathway and reduces cytokine storm
Lambda-carrageenan [43]	Marine algae	- Reduces expression of viral proteins by suppressing viral replication
Terphenyllin Tirandamycin A [29]	Scleractinia associated organisms	- Form hydrogen bonds and dock with M^pro^
Phlorotannins (17 molecules) [30]	*Sargassum spinuligerum* brown algea	- Inhibit Sars-Cov-2 M^pro^ through hydrogen bonding and hydrophobic interactions
Five Marine compounds (C_19_H_40_O_3,_ C_16_H_30_O_2,_ C_22_H_32_O_4,_ C_21_H_26_O_3,_ C_31_H_30_Br_6_N_4_O_11_) [31]	Aplysindae Sponge, soft coral *Pterogorgia citrina* *Petrosia strongylophora* sp.	- Interact with M^pro^ through hydrogen and hydropobic interactions
Phycocyanobilins (PCB) [32,33]	Cyanobacteria, algae rhodophytes	- Inhibits M^pro^ and RNA dependant RNA polymerase - Interact with RBD of spike protein through Vander Waal interactions and hydrogen bonding
Sulfated Polysaccharides [34,35,44,45]	Cyanobacteria, brown algae (Saccharina japonica)	- Binds spike protein and prevents viral entry into cells - Plays an important role in shielding against the virus due to their anionic properties and molecular weight
Bromotyrosines [40,41,46,47]	Marine sponges	- Inhibits protein synthesis, replication, and proliferation of HIV-1 - Binds spike protein and inhibits viral entry

## Data Availability

Not applicable.
